# Effects of Apigenin and Apigenin- Loaded Nanogel on Induction of Apoptosis in Human Chronic Myeloid Leukemia Cells

**DOI:** 10.22086/gmj.v0i0.1008

**Published:** 2018-05-19

**Authors:** Nooshin Samadian, Mehrdad Hashemi

**Affiliations:** ^1^Department of Molecular and Cellular Sciences, Islamic Azad University ,Tehran medical sciences branch, Tehran, Iran; ^2^Department of medical biotechnology, Islamic Azad University ,Tehran medical sciences branch, Tehran, Iran

**Keywords:** Apigenin, Nanogel, Chitosan, Flavonoid

## Abstract

**Background:**

Diet plays an important role in cancer prevention. Apigenin, a flavonoid with thechemical formula C15H10O5 , is abundantly present in vegetables. Vegetarian foods containing flavonoids are rich sources of bioactive compounds. Flavonoids have been utilized in herbal treatment. Nanogels are drug delivery systems based on polymers and are used in tissue engineering and for drug delivery. This study was conducted to compare the effects of apigenin and a nanodrug on the viability of the K562 cell line of chronic myeloid leukemia at different durations under laboratory conditions.

**Materials and Methods:**

Chitosan was first dissolved in 1% acetic acid, and ethylene dichloride EDC and NHS were added to the solution. Then, the nanodrug was prepared by loading apigenin into stearate–chitosan nanogel (scs nanogel), and its physical and morphological characteristics were evaluated by TEM, DLS, and FTIR. Trypan blue staining, MTT assay, and flow cytometry were used to analyze the effects of various concentrations of apigenin and apigenin-loaded chitosan–stearate nanogel (APG–SCS) at 24, 48, and 72 h after they were applied to the K562 cell line.

**Results:**

The diameter of the nanodrug particles was measured using DLS and confirmed by TEM. The K562 cells treated with APG–SCS and with apigenin exhibited significant differences compared with the control (P < 0.05). Apoptosis was detected by flow cytometry.

**Conclusion:**

This study showed that the toxic effects of apigenin and the nanodrug improved with increasing concentrations and exposure durations compared to those in the control.The toxic effect of apigenin loaded into the stearate-chitosan nanogel was greater than apigenin, and the toxic effects of both materials were greater compared to the control under laboratory conditions.

## Introduction

Chronic myeloid leukemia (CML) is a disorder in blood-forming stem cells and is identified by the Philadelphia chromosome. This chromosome is the result of a reciprocal translocation between chromosomes 9 and 22, which is known as t [9; 22] [q34; q11]. The Philadelphia chromosome causes the expression of the BCR-ABL tyrosine kinase, which is an oncoprotein [1]. The treatment of CML includes chemotherapy, radiotherapy, immunotherapy, and bone marrow transplant [2]. Imatinib mesylate is a first-generation tyrosine kinase inhibitor that has been effectively used to treat CML [3].

Despite the positive results obtained by using it in the past, almost 33% of patients with CML are not completely cured by imatinib, and the development of resistance and intolerance to imatinib is still a major challenge [1].

Inhibitors such as doxorubicin and etoposide are the most commonly used chemotherapy drugs for treating leukemia. Unfortunately, severe complications of and resistance to chemotherapy drugs are considerable challenges [4]. Resistance to doxorubicin in patients with leukemia often results in chemotherapy failure [5]. Chemotherapy has been the most common strategy used to treat CML, and the majority of patients recover after a short course of treatment. However, resistance to drugs and disease recurrence are quickly observed in advanced stages of the disease and are a major limitation to the success of treating leukemia [6]. Today, several new compounds and treatment approaches are under study and are used clinically to prevent resistance to imatinib [3]. Combinatorial treatments are thus being investigated, which could increase the effectiveness of standard chemotherapy drugs and reduce the resistance to and the complications caused by chemotherapy drugs [4].

Plant materials have been used for healing wounds in Iran since ancient times. A review of historical manuscripts on medical sciences written by Iranian scientists in the Middle Ages provides valuable information regarding the utilization of medicinal plants and the use of natural materials as drugs. Most of the traditional medications in any country and region are related to their native plants [7-8]. Chemotherapy drugs are primarily expensive and mutagenic, carcinogenic, and teratogenic. The majority of patients suffers from considerable side effects and do not recover because of increased drug resistance. Therefore, new treatment methods are needed for the recovery and survival of patients [2]. Apigenin is found in chamomile (Anthemis cotula), a medicinal plant of the Asteraceae family [9]. The chemical formula of apigenin is 5, 7, 4 -trihydroxyflavone, and it remains primarily unchanged during cooking [10]. Apigenin is abundantly found in vegetables such as celery, parsley, and green pepper; in barley; and in fruits, including oranges and grapefruits [9]. Apigenin is of low toxicity [11] and has strong antioxidant and anti-inflammatory [12], antiproliferative [11-13], apoptotic [13-14], anticancer [14-15], antitumor [11], anti-mutation [14-16], antimetastatic [17], antitumor proliferation and angiogenesis [16], and anti-free radical properties [16]. Nanogels are able to absorb large quantities of water or biological fluids. Swelling, high loading capacity and stability, and biocompatibility are among the suitable properties of nanogels for drug delivery. One of the important properties of nanogels is their inclination to swell because the swelling controls diffusion [18]. Chitosan–stearic acid nanogel is rapidly absorbed by tumor cells, remains in the blood circulatory system for a long time, and can maintain the drug concentration [19]. The aim of the present study was to compare the toxicity caused by apigenin and by the apigenin-loaded nanogel in the K562 cell line.

## Material and Methods

### Chemical Material

Apigenin-(Sigma-Aldrich Co., Kappelweg 1, Schnelldorf, Germany)- chitosan (Sigma Pharmaceutical Company, St. Louis, MO, USA) acetic acid 1% (Merck Pharmaceutical Company Marburger Str.14, Darmstadt, Germany)

Trypan Blue (Bio-Idea-Iran)-DMSO (Sigma Pharmaceutical Company, St. Louis, MO, USA)-PBS (Sigma Pharmaceutical Company, St. Louis, MO, USA)-MTT (Sigma Co., St. Louis, MO, USA)-Pen/Strep (Caisson Laboratories, Inc. 100E, Smithfield, USA)-FBS (Gibco Laboratory, Gaithersburg 9, MD, USA)- RPMI 1640 (Gibco¬ Laboratory, Gaithersburg 9, MD, USA)-Kit Annexin V (IQ Company, Rozenburglaan, Groningen, The Netherlands)-NaHCO_3_ (Merck Pharmaceutical Company Marburger Str. 14, Darmstadt, Germany)- NaOH (Merck Pharmaceutical Company Marburger Str. 14, Darmstadt, Germany)-NHS (Sigma Pharmaceutical Company, St. Louis, MO, USA).

### Preparation of Chitosan–Stearic Acid Aanogel

Half a gram of chitosan was added to a container with 100 ml of acetic acid and dissolved at room temperature using an electric mixer to obtain a uniform and homogeneous 5% (5 mg/ml) chitosan solution. The solution was then sonicated using a sonicator at 70 kHz for 20 min. The pH of the solution was adjusted to 4.0. Then, 36 μmol of stearic fatty acid dissolved in methanol, 90 μmol of ethylene dichloride (EDC), and 55 μmol of N-hydroxysuccinimide (NHS) were slowly added to the 5% chitosan solution and mixed for 2 h. Then, 50 ml of ethanol was poured into the solution and stirred for 3 h in the dark. 10 N sodium hydroxide (NaOH) was then added to the chitosan gel under alkaline conditions, and the solution was mixed at 6000 rpm for 20 min at 4°C, centrifuged, and precipitated. The precipitate was washed first with distilled water and then with ethanol and again with distilled water, dissolved in 100 ml of 1% (v/v) acetic acid, sonicated at 70 kHz for 15 min, and, finally, filtered several times using 0.22-μm syringe tip filters. The chitosan–stearic acid nanoparticle solution in the ratio 1:9, termed as the SI sample, was kept at 4°C [20]. One-to-one ratio of apigenin and stearate-chitosan nanogel was sonicated using a sonicator at 60 kHz for 15 min and then incubated at 15°C to load the apigenin into the nanogel.

### Evaluation of the Physicochemical Properties of the Chitosan Nanogels

Dynamic light scattering (DLS), transmission electron microscopy (TEM), and Fourier-transform infrared spectroscopy (FTIR) techniques were used.

### DLS

Zeta potential and DLS were used to determine the mean size and size distribution of the nanogel and the nanodrug particles. The mean sizes of the nanogel and the nanodrug particles were determined by DLS after 229 and 230 counts, respectively, and the polydispersity index (PDI) was calculated.

The samples were placed in a water bath sonicator and then in the special cuvette of the instrument, and then the diameter of the particles, the diffusion rates of the nanogels, and also the zeta potentials were calculated. Zeta potential is a measure of particle surface charge in a specific liquid medium, and it is measured by electrophoretic light scattering that measures the electrophoretic mobility of the particles caused by the application of an electric field.

### TEM

TEM is a microscopy technique in which an electron beam is transmitted through an ultrathin sample to form an image of the interactions between the electrons and the sample. This imaging is carried out at a much higher resolution compared to that of light microscopes. This test was performed using a CM 120 model TEM (manufactured by the Philips Company) at 20°C in the 31X-680KXmeasuring range. (Magnification:45K)

### FTIR

In nanotechnology, the FTIR method is used to confirm whether a specific species is functionalized or not functionalized with another compound and/or to confirm the accurate coating of a surface with specific compounds.

Infrared waves are of low energy levels and cannot break bonds or cause electron transfers. Each frequency represents the presence of a specific functional group in the sample. Therefore, useful information can be obtained regarding the molecular structure of organic compounds.

In this study, spectral analysis was performed for chitosan (CS), stearic acid, stearate–chitosan nanogel (SCS nanogel), and apigenin and apigenin-loaded chitosan–stearate nanogel (APG–SCS) using FTIR by employing a Thermo Nicolet Nexus 870 FT-IR ESP spectrometer (manufactured in the USA).

### Cell Culture

The K562 cell line of CML was purchased from the National Cell Bank of Iran, and the cells under study were cultured in a 25-ml flask for 24 h before being treated with the drug or the nanodrug. The studied cells were cultured in RPMI-1640 medium containing 10% fetal bovine serum (FBS) and 1% penicillin and streptomycin solutions in an incubator (VS-9160C; Bionex, Seoul, South Korea) with a 5% CO2 atmosphere at 37°C [14].

### Assessment of Cell Viability

Trypan blue staining and MTT assay were used to assess the cell viability, and flow cytometry was used for determining the degree of apoptosis.

### Trypan Blue Staining

On the first day, 10^5^ cells were added to each well of the culture containers and the plates were incubated under 5% CO_2_ atmosphere at 37°C for 24 h. The apigenin (the sham shows the result of treating the cells with DMSO; the amount of DMSO in the sham is the same as the concentration at which the drug is dissolved) together with the nanogel and the apigenin-loaded nanogel (the sham control shows the result of treating the cells with DMSO together with the nanogel, and the same concentration as used) solutions at 2.5, 5, 10, 20, 50, and 100 μmol/ml were added to each well of the plates, respectively. After 24, 48, and 72 h of adding the drug to the cells, a determined volume of the cell suspension at each concentration was taken, and 10 μl of 0.4% trypan blue was added to it, and after 5 min, the blue cells (dead cells) and the hyaline cells (living cells) were counted using Neubauer counting chambers [21].

### MTT Assay

The tetrazolium-based MTT (3-(4,5-dimethylthiazol-2-yl)-2,5-diphenyltetrazolium bromide) assay was performed in 96-well plates, in which the yellow tetrazolium was reduced by the mitochondrial enzyme succinate dehydrogenase in living cells and purple formazan crystals were formed. Each well in the plates received 1.5×10^4^ cells, and the plates were incubated under 5% CO2 atmosphere at 37°C for 24 h. The cells were treated with apigenin (the sham shows the result of treating the cells with DMSO; the amount of DMSO in the sham is the same as the concentration at which the drug is dissolved, and the same concentration was used) and apigenin-loaded nanogel (the sham shows the result of treating the cells with DMSO together with the nanogel, and the same concentration was used) at 2.5, 5, 10, 20, 50, and 100 μmol/ml and stained with the MTT solution after 24, 48, and 72 h. After 3–5 h of incubation at 37°C, 100 μl of dimethyl sulfoxide (DMSO) (pure solution; Sigma-Aldrich) was added to each well, the plates were placed on a shaker for 10–15 min, the light absorption was read at 570 nm by an ELISA microplate reader (ELx800, BioTek, Winooski, VT, USA), and the cell viability was calculated using the following formula [14]:

1 Cytotoxici% = 1- (Mean absorbance of toxicant) / (Mean absorbance of negative control)×100

2 Viability% = 100 − Cytotoxicity%

### Flow Cytometry

Using IQ Annexin kits (IQ Company, Rozenburglaan, Groningen, The Netherlands), about 10^6^ cells were suspended in Annexin V buffer for 72 h at different concentrations of apigenin and apigenin-loaded nanogel to analyze apoptosis [14].

### Statistical Analysis

GraphPad Prism 6 was used for statistical analysis, and the data were expressed in ±SD. One-way ANOVA was performed for comparison of the mean values, and P-values <0.05 were considered as statistically significant.

## Results

### DLS

**Figure-1:**
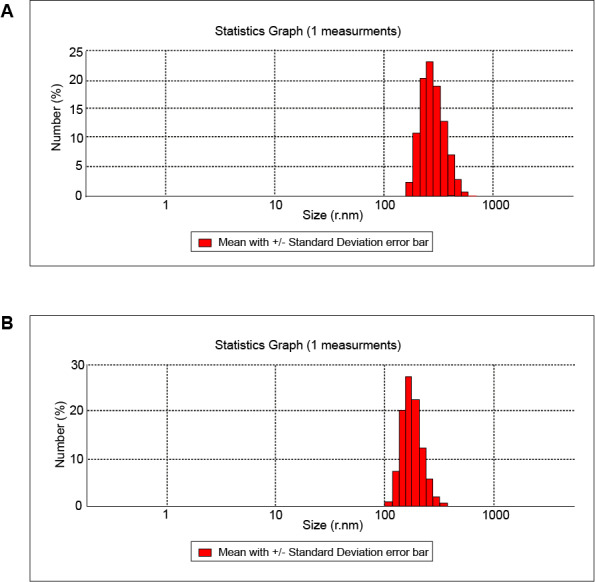
Figure of Statistics Showing Results Related to the **(a)** Nanogel **(b)** Apigenin loaded chitosan- Stearate Nanogel

**Figure-2:**
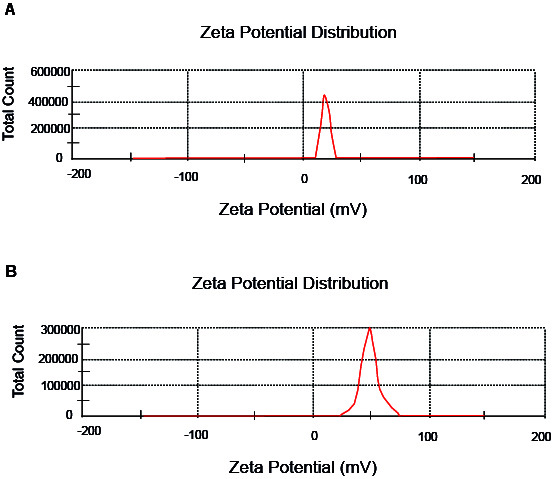
Zeta Potential Distributions for the **(a)** Nanogel **(b)** Apigenin loaded chitosan-Stearate Nanogel

Table-1 shows the results of DLS for the nanogel and the nanodrug. The sizes of the nanogel and the nanodrug particles analyzed by DLS are depicted in Figure-1. The average diameters of the nanogel and the nanodrug particles were 265 and 194.6 nm, respectively. The zeta potentials of the samples were determined at 25°C and are listed in Table-1 and depicted in Figure-2. The nanogel particle size distribution was also confirmed by TEM. The PDI values obtained for the nanogel and the nanodrug were 0.289 and 0.482, respectively.

**Table-1:** DLS of Chitosan -Stearate Nanogel and of the Nanodrug

**sample**	**Nanogel(chitosan-stearate nanogel)**	**Nanodrug(AP-SCS)**
**Mean Particle size by DLS (nm)**	265	194.6
**DLS record number**	229	230
**Zeta potential (mV)**	19.4±3.21	47.7±3.7
**PdI**	0.289	0.482

**Nanodrug(AP-SCS)** (apigenin loaded chitosan -Stearate nanogel) **DLS** (Dynamic Light Scattering) **PdI** (poly dispersity index)

### Transmission Electron Microscopy (TEM)

**Figure-3:**
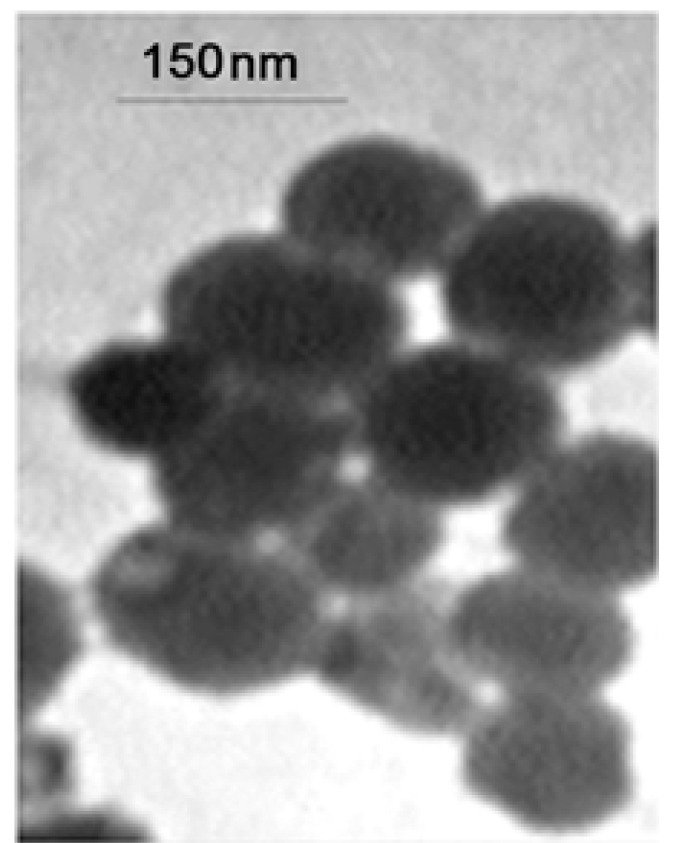
Image of Nanodrug Particles Taken by TEM (Further Details Presented in the text)

Figure-3 depicts the TEM results showing the size and shape of APG–SCS chitosan–stearic acid. The apigenin-loaded nanogel exhibited a smooth surface in the TEM image, and the nanoparticle size was 150 nm.

### FTIR

**Figure-4:**
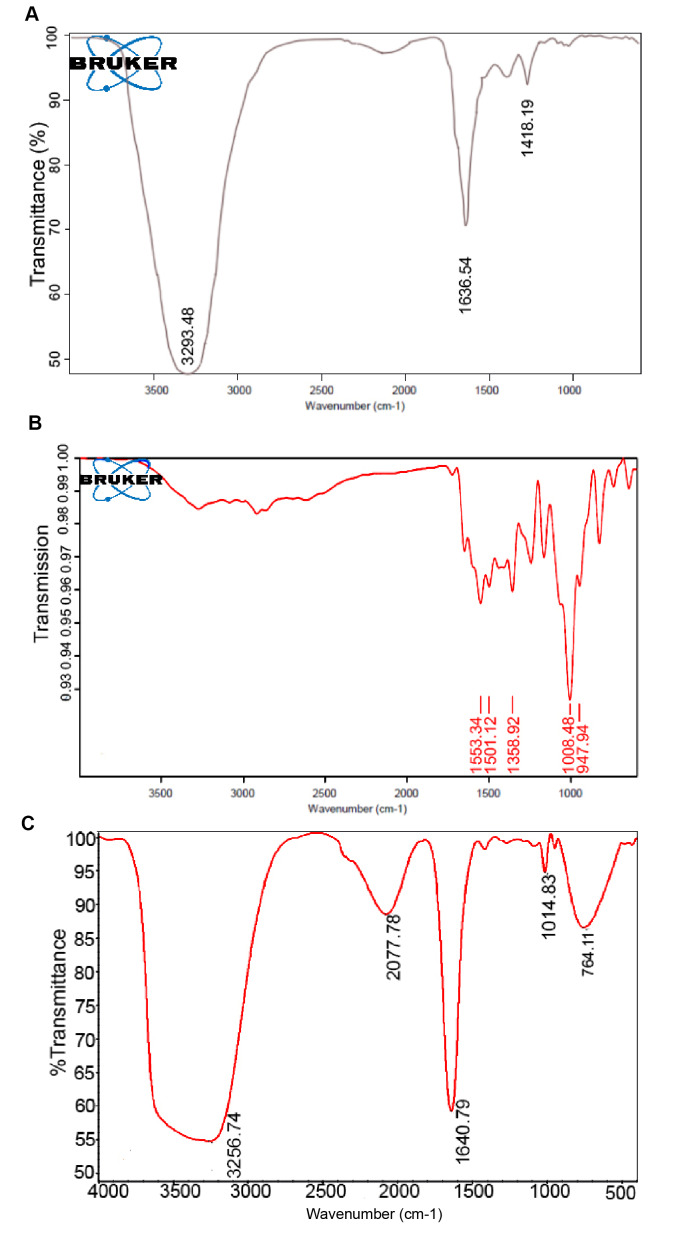
FTIR Spectra of Nanogel **(a)** Apigenin **(b)**Apigenin loaded chitosan- Stearate Nanogel **(c)**

The FTIR results of evaluation of the structures of chitosan–stearic acid nanogel, apigenin, and apigenin-loaded chitosan–stearic acid nanogel (APG–SCS) are shown in Figure-4.

The most important bands in the apigenin FTIR absorption spectrum in the 1501 and 1553 cm^-1^ regions were related to the frequency of the C-C and C=C tensile vibrations and shifted to the 1640 cm^-1^ region in the APG–SCS stearate-chitosan nanogel absorption spectrum and were intensified.

The apigenin absorption band in the 1947 and 1008 cm^-1^ regions also shifted to the 1014 cm^-1^ region in the APG–SCS nanodrug absorption spectrum. The presence of the extra band in the 2077 cm^-1^ region in the nanodrug absorption spectrum and its absence in the apigenin and SCS nanogel absorption spectrum are related to a small component of C-H present in the ring with a reduced degree of freedom in motion. Moreover, the strong peak in the 1553 cm^-1^ region in the apigenin absorption spectrum is related to vinyl spectrum with double bonds, but it was completely eliminated in the nano-apigenin absorption spectrum. This result confirms the formation of apigenin-bearing nanogels. The sharp peak related to the tensile vibration frequency of O-H in the nanogel in the 3293 cm^-1^ region was observed in the form of a broad peak in the APG–SCS nanodrug absorption spectrum in the 3256 cm^-1^ region, whereas this peak was not observed for apigenin. This finding is related to the position of apigenin inside the nanogel polymer and to the proton exchange between the nanodrug and the solvent.

### Trypan Blue Staining

**Figure-5:**
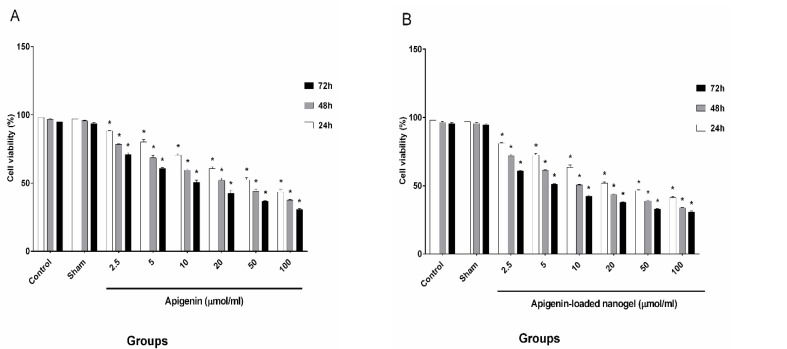
Results of Trypan Blue on Cancer Cells at Various Concentrations

The results indicating the viability of cells treated with six selected amounts (doses) of apigenin and of the nanodrug, which were evaluated by trypan blue staining, are demonstrated in Figure-5.

The cells were counted, and the IC50 values for the different concentrations of apigenin and the nanodrug were as follows: IC50s (24h:50 ± 1.7 µmol/ml, 48h:20 ± 1.66 µmol/ml, 72h:10 ± 1.48 µmol/ml) and IC50s (24h:20 ± 1.07 µmol/ml, 48h:10 ± 0.53 µmol/ml, 72h:5 ± 0.51 µmol/ml), respectively.

### MTT Assay

**Figure-6:**
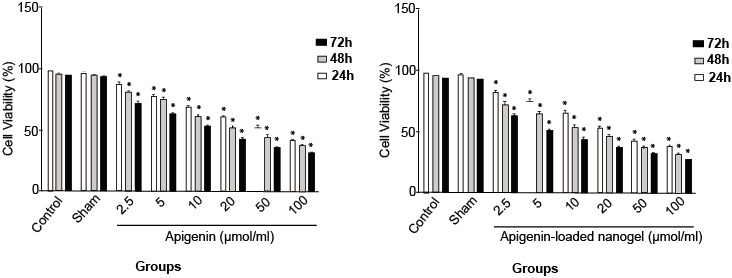
Results of Mtt Assay on Cancer Cells at Various Concentrations

Figure-6 shows the results of the viability of cells treated with six selected amounts (doses) of apigenin and of the nanodrug, which were evaluated by MTT assay. The cells were counted, and the IC50 values for the different concentrations of apigenin and the nanodrug were as follows: IC50s (24h:50 ± 1.79 µmol/ml, 48h:20 ± 0.82 µmol/ml, 72h:10 ± 0.92 µmol/ml) and IC50s (24h:20 ± 1.07 µmol/ml, 48h:10 ± 1.98 µmol/ml, 72h:5 ± 0.65 µmol/ml), respectively.

### Flow Cytometry

**Figure-7:**
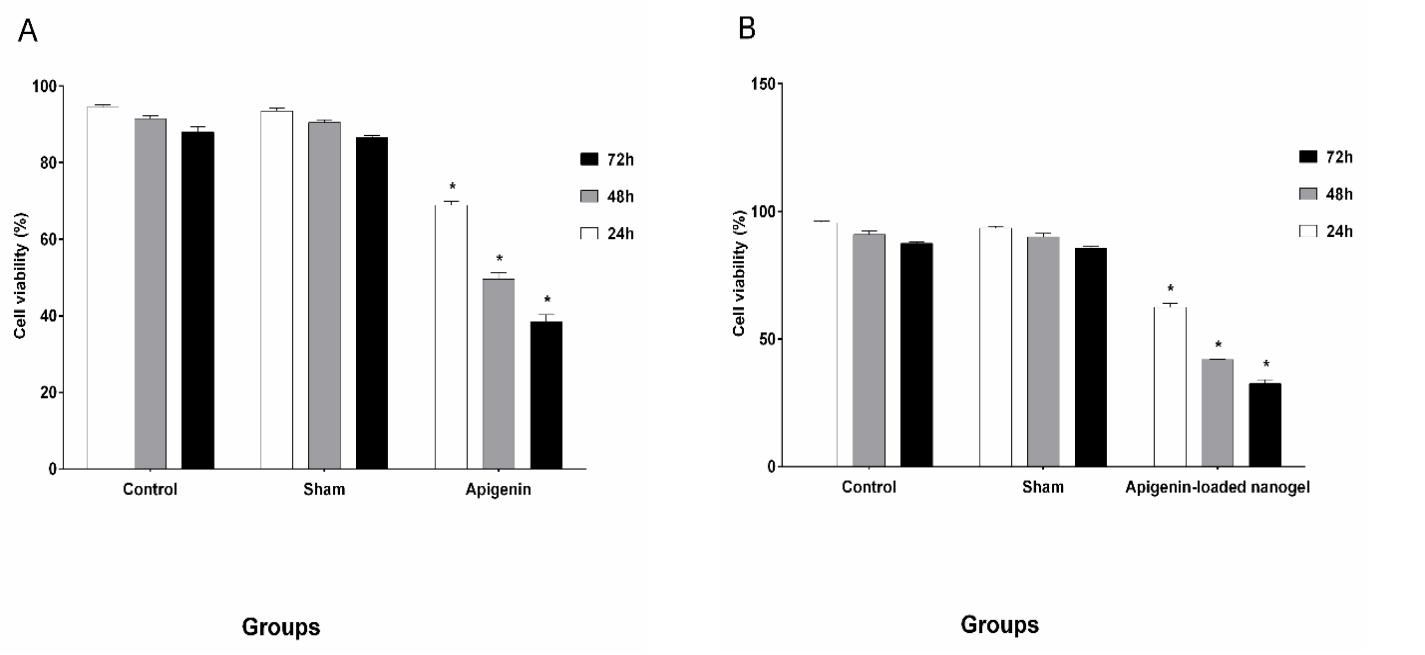
Results of Flow Cytometry on Cancer Cells at Various Concentrations

The degrees of apoptosis in the K562 cell line after treatment with 20 and 10 μmol/l concentrations of apigenin and apigenin-loaded nanogel, evaluated using flow cytometry, are presented in Figure-7.

**Figure-9:**
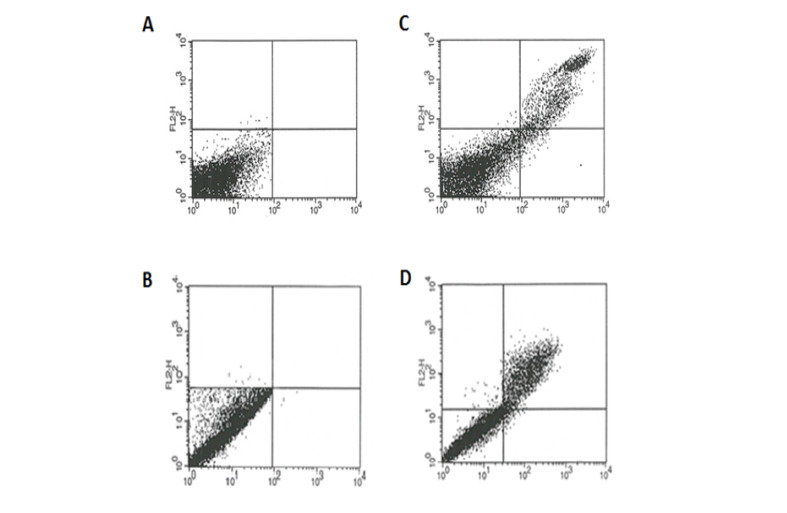
Expression of Phosphatidylserine Molecules on the Surface of K562 Cell Line of Chronic Myeloid Leukemia in 48h **A)** Control **B)** Treatment by Apigenin 20μm/ml **C)** Control **D)** Treatment by Apigenin loaded chitosan-Stearate Nanogel 10μm/ml

The degrees of apoptosis for apigenin after the exposure durations of 24, 48, and 72 h were 30.34%, 51.54%, and 60.21%, respectively, while the corresponding values for the nanodrug were 38.81%, 57.84%, and 66.37%, respectively (Figure-9).

## Discussion

CML is a stem cell disorder characterized by the presence of the Bcr-Abl gene. Abnormal expression of the Bcr-Abl gene and excessive activity of protein tyrosine kinases are considered as the causal agents of CML [6]. Moreover, about one-third of deaths caused by cancer can be prevented by adopting suitable diets [22].

Despite the advances that have been achieved and the effectiveness of treatments, mortality due to leukemia still persists [2]. In recent years, nanotechnology has been widely used for drug delivery. Improved solubility of hydrophobic drugs and their increased accumulation in tumors are among the advantages of these delivery systems [18].

In the late 1990s, drugs used to treat cancer have been shown to affect both normal and abnormal cells. Therefore, chemotherapy drugs were also found to be cytotoxic to normal cells. Targeted therapies do not influence normal cells because they employ methods that affect the control of cancer cell growth, division, and expansion. Advances in targeted therapies have led to improvement of their results in treating patients with leukemia [23].

Paelega *et al*. observed that the drug was present inside the liposome polymer dipalmitoylphosphatidylcholine (DPPC) by forming hydrogen bonds between the hydroxyl group in apigenin and the polar heads of the lipids in the C-O-P-O-C sections [24].

Oligosaccharide chitosan–stearic acid (COS-SA) rapidly penetrates into cancer cells by producing micelles [25]. The hydrophobic polymer derived from stearic acid (CSO–SA) has been used for gene transfer into cells [19]. In the last decade, there has been a continuous increase in studies evaluating new strategies for treating cancer and for utilization of natural compounds [26]. Nanogels are one of the controllable drug delivery systems, especially for delivering bioactive materials, owing to the unique and special properties of hydrogels. Nanogels can coat various types of polyphenols [18].Using a combination of chemotherapy drugs and bioactive compounds such as polyphenols can be considered as one of the strategies. A combination of doxorubicin, etoposide, and apigenin induces synergistic effects in reducing the ATP levels, increases the consequent apoptosis, and stops the cell cycle [4]. Under laboratory conditions, apigenin inhibits doxorubicin-induced mutagenicity.

Moreover, apigenin reduces the in vivo mutagenic effects of doxorubicin and cyclophosphamide [27]. One of the changes in the early stages of apoptosis is the transfer of phosphatidylserine from the inner section of the plasma membrane to the outer layer and the appearance of phosphatidylserine (PS) on cell surfaces. This method is based on the combination of the calcium-dependent binding protein Annexin V with membrane phospholipids (PS). Necrotic cells also offer PS molecules on their surfaces and lose their membrane integrity. Therefore, these two stages can be distinguished by adding propidium iodide (PI) to Annexin V [14].

PI only changes the color of DNA in necrotic cells [14]. A study on the effect of apigenin loaded into nanoparticles (PLGA) on skin cancer indicated that apigenin-loaded nanoparticles were more effective than apigenin because of their smaller size and faster motility [28].

In the present study also, the apigenin-loaded nanogel was more capable of destroying the K562 cells compared to that by apigenin. Nano-encapsulated apigenin has been successfully used as a substitute for treatment with corticosteroids and in curing eczema without side effects [29]. Activation of caspase 3 resulting from the effect of PLGA–apigenin nanoparticles on human skin cancer cells confirms the superior apoptotic activity of apigenin-loaded nanoparticles compared to that of apigenin [30]. In the present study also, it was observed that the apigenin-loaded nanogel exhibited superior apoptotic activity compared to that of apigenin. Apigenin-loaded nanoparticles encapsulated by PLGA were shown to induce mitochondrial apoptosis in skin cancer [28]. Liu *et al*. (2014) reported that apigenin killed cancer cells in cervical cancer in a dose-dependent manner [31]. In the present study also, the toxicity caused by apigenin and the nanodrug in K562 cells was dose- and exposure-duration-dependent. Ren also showed that apigenin suppressed the proliferation of various cells in lung cancer in a dose-dependent manner [32]. The present study also showed that the effect of apigenin on the viability of K562 cells improved with increases in concentration and exposure duration and was also dose-dependent. In 2011, researchers showed that apigenin was toxic to myeloma cells but not to normal blood cells. Apigenin suppresses the proliferation of cells in multiple myeloma by inducing apoptosis [33]. Treating normal human peripheral blood cells with apigenin did not cause sensitivity to apigenin. The effects of apigenin on normal human fibroblasts did not cause their activity to stimulate apoptosis, and the normal cells remained healthy [16] After intraperitoneal injections in rats, no statistically significant differences were observed between the control groups and those that received pure DMSO.

Therefore, DMSO did not influence the results [34]. Solmaz *et al*. studied the effects of apigenin on K562 cells and on cells resistant to imatinib [3]. The present study also studied and compared the effects of apigenin and apigenin-loaded nanogels on the K562 cell line derived from a CML patient.

Solmaz *et al*. found that apigenin toxicity against K562 cells was dose- and time-dependent; they also determined the cytotoxic effects of apigenin on K562 cells using the MTT assay and reported 16 mmol IC50 value after 48 h. In the present study also, the effects of apigenin on K562 cells was investigated, and the observed IC50 value after 48 h was 20 mmol, and the IC50 value for the apigenin-loaded nanogel was 10 mmol. In addition, apigenin toxicity against K562 cells was found to be dose- and time-dependent. The effect of nanoparticles on the vital capacity of cells was approximately two times at the same concentration and time. The greater effectiveness of the nanoparticle was due to the presence of the nanogel in which the drug is loaded. The nanomedication causes the IC50 value of the nanoparticle to reduce or decrease by about half the amount of IC50 value of the drug. Hashemi *et al*. [14] investigated the acute lymphoblastic leukemia cell line and, using flow cytometry, reported a 19% degree of apoptosis caused by apigenin after 24 h. In the present study also, the degrees of apoptosis resulting from the use of apigenin and apigenin-loaded nanogel after 24 h were 30.34% and 38.81%, respectively.

**Figure-8:**
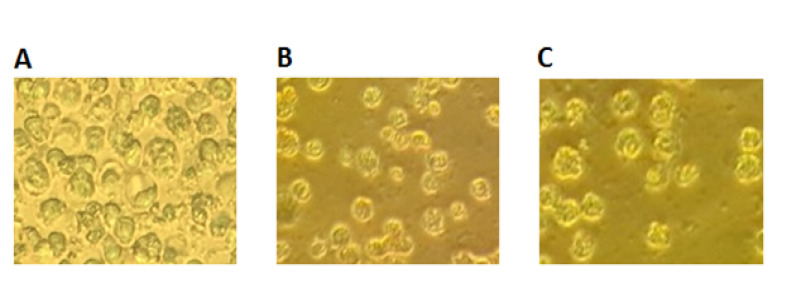
Photomicrograph of K562 Treatment in 48h: A) Control B) Treatment by apigenin 20μm/ml. C) Treatment by Apigenin loaded chitosan- Stearate Nanogel 10μm/ml ×20

Furthermore, apoptosis of K562 cells was induced by apigenin and the nanodrug, and no toxic effect was observed in normal blood cells [35]. The results obtained from trypan blue staining and MTT assay showed that after 24 h, viability of cells treated with apigenin was 50µmol/ml and that for celles treated with apigenin loaded nanogel was 20µmol/ml, and after 48 h, these levels changed to 20 and 10 µmol/ml (Figure-8), whereas after 72 h, the viability declined substantially to 10 and 5 µmol/ml, respectively. The viability of K562 cells in the presence of apigenin and apigenin-loaded nanogel was dependent on the dose and the exposure time, and the degree of apoptosis in cells treated with apigenin-loaded nanogel was much higher than that in cells treated with apigenin. The sham group showed no significant difference compared with the control group and had no effect on the cells and results.

The effect of apigenin and apigenin-loaded nanogel in chitosan–stearic acid nanogel was studied in the K562 cell line of CML. The results indicated that compared with the control group, the toxic effect of both materials on these cells increased with longer exposure times, and the extent of this increase depended on apigenin and APG–SCS concentrations, and the cell viability decreased with increases in the concentrations of these materials (one-way ANOVA, P < 0.05). Moreover, at constant exposure duration and concentration, the apigenin-loaded nanogel was much more effective than apigenin, and both exhibited significant differences when compared with the control group. This study was conducted for the purpose of clinical treatment for determining whether it may be possible to use the nanodrug together with chemotherapy drugs or as a substitute for chemotherapy drugs.

## Conclusion

Apigenin and the apigenin-loaded nanogel substantially suppressed the growth of K562 cells, and this suppression was dose- and exposure-time-dependent, which was in agreement with rsults of previous research. In the apigenin-loaded nanogel, the dose used was reduced. This nanogel (chitosan–stearate nanogel) can be of interest as a new strategy for cancer treatment.

## Conflict of Interests

Authors have no conflict of interests.
